# Cell phone-supported cognitive behavioural therapy for anxiety disorders: a protocol for effectiveness studies in frontline settings

**DOI:** 10.1186/1471-2288-11-3

**Published:** 2011-01-10

**Authors:** Joakim Ekberg, Toomas Timpka, Magnus Bång, Anders Fröberg, Karin Halje, Henrik Eriksson

**Affiliations:** 1 Department of Medical and Health Sciences, Linköping University, Linköping, Sweden; 2 Department of Computer Science, Linköping University, Linköping, Sweden; 3 Unga Vuxna Clinic, Östergötland County Council, Linköping, Sweden

## Abstract

**Background:**

Reviews of randomized controlled trials (RCTs) of cognitive behavioural therapy (CBT) for anxiety disorders have reported large pre- to post-treatment within-group effect sizes on measures of anxiety when supplied in therapist consultations and in technology-supported settings. However, the stringent experimental control of RCTs results in a lack of external validity, which limits the generalizability of findings to real-world frontline clinical practice. We set out to examine the specification of a protocol for study of the effectiveness of cell phone-supported CBT for in situ management of anxiety disorders.

**Methods and design:**

Nominal group methods were used for requirements analysis and protocol design. Making a distinction between different forms of technology-supported therapy, examination of therapists' role, and implementing trials in existing organizational and community contexts were found to be the central requirements in the protocol.

**Discussion:**

The resulting protocol (NCT01205191 at clinicaltrials.gov) for use in frontline clinical practice in which effectiveness, adherence, and the role of the therapists are analyzed, provides evidence for what are truly valuable cell phone-supported CBT treatments and guidance for the broader introduction of CBT in health services.

## Background

Ample evidence indicates that anxiety is under treated in western societies, and that large numbers of the population suffer needlessly. The World Health Organization (WHO) has estimated that 40% of disability attributed to anxiety reflects the fact that many potential anxiety patients never reach health care [1]. A major factor contributing to this shortcoming is that evidence-based psychotherapies are not practiced widely in community settings [2,3]. For instance, less than a quarter of those with anxiety disorders in the United Kingdom receive treatment of any sort [4]. In the United States, only about 40% of patients with mood or anxiety disorders receive any treatment [5]. It is in this context that expectations of cognitive behavioural therapy (CBT) are high. CBT methodology has the advantage of using well-defined treatment practices that can be easily taught to a variety of therapists and whose implementation can be monitored. Reviews of randomized controlled trials (RCTs) of CBT for anxiety disorders have reported large pre- to post-treatment within-group effect sizes on measures of anxiety when supplied in therapist consultations [6,7], in computer-supported sessions at practices [8,9], and over the Internet [10,11]. In Sweden, CBT is the current treatment of choice for mild to moderate anxiety disorder [12].

Despite its proven efficacy, CBT still seems to be neglected in practice settings. In this study, we address 2 possible reasons for this situation. The first is that most of the research has been conducted using samples with isolated (rather than comorbid) disorders, but most therapists help individuals with multiple comorbid problems. It is not known if CBT techniques adapted to a particular client's needs by a skilled therapist in a community setting achieve a better result than a therapist following a structured routine. It does seem that experienced therapists prefer to select from a variety of techniques rather than to follow a regimented program. Moreover, patient non-adherence is a persistent and complex phenomenon in frontline CBT practice [13]. The refractory nature of this problem is reflected by the fact that a large proportion of non-adherence has come to be routinely accepted when planning interventions. Critics argue that RCTs' stringent experimental controls (e.g. patients with homogenous diagnoses and highly trained and supervised therapists) result in a lack of external validity, which limits the generalizability of findings to real-world or frontline clinical practice [14,15]. In addition, studies of the effectiveness of CBT for anxiety disorders have consistently reported lower patient adherence than RCTs [13,16].

**Figure 1:**
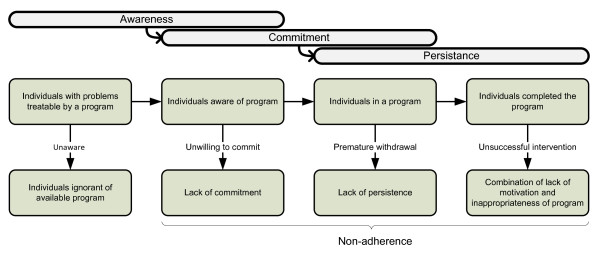
**Health service perspective on non-adherence to CBT for anxiety disorders**.

The second possible reason for under utilization is that a fundamental principle in CBT is to document and adjust behaviour and thought processes when and where they occur. Accordingly, a goal in developing novel applications of CBT should be to assist therapy in situ, that is, exactly when it is needed. Using participatory design methods, we have developed a set of such applications [17]. In this process, we acknowledge that adherence to mental health services is no longer only a matter of complying with a decided course of treatment in a clinical setting, but of reaching, connecting to, motivating, and sharing health decisions with patients and populations (Figure 1). The perspective on health service provision thereby shifts from biomedicine to infomedicine; patients and health workers join in informed, shared decision-making and governance [18].

We conclude that there is a need to examine carefully the external validity and effectiveness of CBT treatments for anxiety that have already been shown to be efficacious, and to identify factors that reduce non-adherence. In this study, we used these presumptions to plan for the examination of a new generation of CBT programs. With this understanding, we set out to define a protocol for examination of the effectiveness of cell phone-supported CBT for anxiety disorders in frontline settings that also allows to evaluate adherence to therapy. Specifically, the aims of this study were to define a protocol for assessment of

• the superiority of cell phone-supported CBT (CBT-cell phone) treatment against anxiety disorders compared with CBT treatment as usual (CBT-TAU), and

• adherence with CBT-cell phone compared with CBT-TAU and CBT provided with access to a placebo technical device (CBT-placebo).

## Methods and design

**Figure 2:**
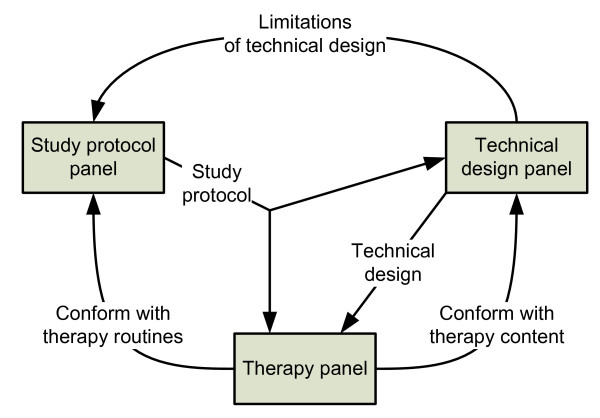
**Flow of study protocol specification and development of requirements during the formulation process**.

Nominal group methods [19] were used for requirements analysis and protocol design (Figure 2). Two expert panels examined requirements on the data to be collected: a technical design panel (*n *= 5) with a background in computer science and cognitive science, and a therapy panel (*n *= 4) consisting of practising therapists. The requirements data collected were transferred to a study protocol design panel (*n *= 6) with a background in social medicine, general practice, CBT practice, health informatics, and cognitive science.

### Requirements data collection

The task communicated to the therapy panel was to make sure the protocol conformed to organizational and therapeutic routines and content. The task communicated to the technical design panel was to make sure that the protocol conformed to present cell phone technology and limitations. In addition, the technical design panel, in cooperation with the therapy panel, developed a specification of the technical systems to ensure that, in accordance with the protocol, the content of the technical solution was compatible with the paper-based homework used in present CBT for anxiety syndrome.

### Requirements data analysis

The data from the study requirement analysis were transferred to a protocol specification by the study protocol panel. The specification was developed during a series of meetings lasting for 2 hours each (*n *= 8) at which the study protocol, technical design, and therapy methods in use were systematically developed and discussed. The specification was finally refined at separate meetings with the therapy and technical design panels.

### Study protocol requirements

The results of the requirement analysis yielded 3 requirement areas that were found to be important when studying the effectiveness and adherence to cell phone-supported CBT in a community context.

### Effectiveness trials

Even if different forms of CBT have been shown to be efficacious in RCTs, therapy provided in a community setting entails a wider variety of patient problems and comorbidity. A recent meta-analytic review of CBT supported by or delivered through information technology (IT) found no significant differences between face-to-face and computer-aided psychotherapy for anxiety and no differences between the system used (PC, palmtop, other) [20]. However, many studies of computer-aided CBT have used small samples. It is also a concern that most studies of computer support in CBT lack details on therapist contacts, time spent on assessment and diagnosis, and sparse reports of interventions delivered by ordinary therapists [21]. When introducing cell phone support in therapy in front-line settings, it is therefore important to be specific regarding what is compared, and to provide a large enough sample to be able to determine differences in effectiveness.

### The role of the therapist

Another important issue in effectiveness evaluations of IT-supported therapy is non-adherence. In RCTs of computer-supported CBT, different methods have been used to deal with dropouts (e.g. including only completers) [20]. Based on the evidence from the RCTs reported, it is tempting to meet the growing demand for CBT with computer-based therapy programs. However, although effective computer-based treatments have been tailored for specific and well-diagnosed patients, in practice there is a substantial comorbidity between anxiety and other mental illnesses, and the therapists variable in this context is still not well understood [21,22]. Tailoring of Internet therapies according to diagnostic profiles may be possible; however, visitors to therapy web sites who have registered on their own initiative have also had larger dropout rates than that reported in RCTs [23].

### Organizational and community context

To examine the effectiveness rather than the efficacy of CBT, it is important to include comorbid disorders and base the study on total populations. Reviews of RCTs of CBT for anxiety disorders have reported large pre- to post-treatment within-group effect sizes on measures of anxiety when supplied in therapist consultations in computer-supported sessions at practices [8,9] and on the Internet [10,11]. Yet, effective dissemination of evidence-based intervention to community stakeholders has been shown to be challenging [24]. Facing the difficulties of closing the gap between research and practice, the UCLA/RAND NIMH Center has developed a framework to assess the relevant contextual factors, identify, develop and evaluate new strategies, provide useful formative feedback, develop capacity among stakeholders and to meaningfully generalize findings. Evidence on dissemination and implementation in an organizational and community context rests on process evaluation of contextual factors, stages of diffusion, and outcome of interventions [24]. A successful outcome in one implementation is only anecdotal evidence, unless some evaluation of the context (structure and process) and adaptations made for the context are also documented.

### Study protocol

The final protocol (NCT01205191 at clinicaltrials.gov) informed by the data analysis was developed with regard to the observed limitations of earlier studies of computer-supported CBT; e.g. the substantial dropout rates, partial neglect of therapist involvement, and lack of direct comparisons between face-to-face and IT-supported methods [21]. The protocol is presented as it is implemented in a study at the Unga Vuxna (Young Adults) clinic in Östergötland County, Sweden.

### Study setting

**Figure 3:**
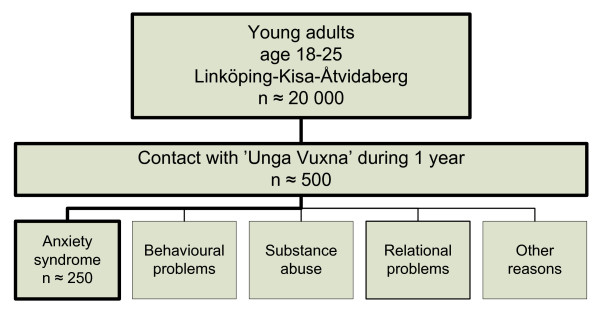
**Overview of the population offered the Unga Vuxna service in Östergötland, Sweden**.

The Unga Vuxna service accepts clients aged 18-25 years from a total population in a region in southern Sweden (*n *= 20,000) following a therapist telephone triage (Figure 3). All therapists (clinical psychologists, social workers) have a two-year (certified) academic training in CBT. The intervention at Unga Vuxna consists of 6 CBT sessions, each lasting 45 minutes. Sessions follow a predefined program, but are also customized to the patient's specific needs, including initial assessment whether the patient has expressed any suicidal intent. Clients also continue to receive pharmacotherapy if previously prescribed by their physician. A report of the patient's progress, including whether the patient has expressed any suicidal intent, is recorded. Homework projects are generated for each patient, to be accomplished before the next session. A report of the patient's progress is recorded and all clients are followed up by a visit at the clinic 3 months after the therapy has been concluded.

### Study inclusion criteria

The inclusion criteria are: (1) age 18 to 25 years and (2) scoring 7 or higher on the anxiety section of the Hospital Anxiety and Depression Scale (HAD) at initial evaluation. Clients are excluded if they: (1) display symptoms at the initial evaluation that suggest referral to a psychiatric specialist, e.g. active suicidal ideas or symptoms of a psychotic disorder, organic mental disorder or alcohol and/or drug dependence or (2) are unable to read, write or speak Swedish.

### Study performance

#### Treatments

Clients receive whatever treatment their therapist prescribes. To replicate natural conditions, interventions received by clients are not constrained by the study. The interventions thus also include, for example, provision of practical/social support at the employment office, and further medical investigations.

#### Procedures

**Figure 4:**
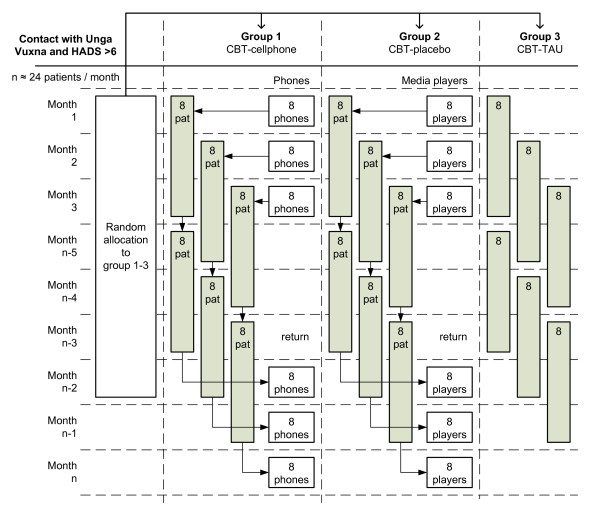
**Study procedures**. The average treatment period at Unga Vuxna is 3 months.

The procedure follows the intention to treat (ITT) principle, that is, based on the initial treatment intent, not on the treatment eventually administered. The Unga Vuxna service accepts clients following a therapist telephone triage. Recruitment to the study is administered in association with the first CBT session at the clinic. At this session, routine screening is performed by administrating the Hospital Anxiety and Depression Scale (HADS) to clients. Individuals found fitting the study criteria are presented with information materials about the study by a study nurse. The materials are made available to the client for evaluation in private without the therapist being present. The allocation schedule is generated by a biostatistician outside the clinic before the study commences using an individual unit of randomization. Individuals who fit the inclusion criteria and have consented to participate are stratified according to whether they are prescribed medication or not (yes, no) and with regard to their HADS-D score. They are then randomly allocated to receive CBT-cell phone, CBT-TAU, or CBT-placebo (Figure 4). To parallel the IT-lending situation in the CBT-cell phone strand (in adherence analyses), the clients allocated to CBT-placebo are provided with an MP3-player with anti-stress materials to use between the sessions. The sealed envelopes are opened in strict numerical sequence by the study nurse. The nurse is instructed to spend no more than 10 minutes before and after each session with each client.

#### Study outcome measures

The primary outcome measure is the Beck Anxiety Inventory [BAI]. The BAI [25] is a 21-item symptom checklist rated on a 4-point scale (0-3). The main secondary set of outcome measures describes patient adherence. Data on adherence are collected in 4 dimensions from the therapists after the last session; that is whether the client has

• discontinued the CBT program by not showing up at sessions;

• discontinued the CBT program by actively informing the therapist;

• been discontinued from the treatment by the therapist; and

• not fulfilled the therapeutic contract, e.g. by not completing homework agreed upon.

The remaining secondary outcome measures are HADS-A and -D, and the General Health Questionnaire [GHQ-12]. The outcome measures are administered on each of 3 occasions: pre-treatment, when the participants had completed their programme, and with follow-up at 6 months. The GHQ has demonstrated validity with young adults (17+ years) from the United Kingdom [26]. The sensitivity and specificity for both HADS-A and HADS-D are similar to the sensitivity and specificity achieved by the GHQ and they perform well in assessing the symptom severity of anxiety disorders and depression in both somatic, psychiatric and primary care patients, and in the general population [27].

### Statistical analysis

Power calculations were performed to determine the minimum sample size needed to fulfil the research aims. The calculations were based on change scores (pre- to post-treatment) between groups in 2 previous studies comparing CBT as usual with CBT supported by handheld computers [28] and CBT provided by stationary surgery-located computers [9]. To detect a standard deviation difference of 0.3 in BAI change scores at 80% power and with 0.05 alpha, we determined that a total sample size close to 150 per stand (450 in total) was needed.

For each of the outcome measures, a series of mixed effects models will be considered. Such models are described in detail by Everitt and Pickles [29]. Model adjustments will be used to analyse associations between pre- and post-randomization values of outcome (i.e. post-CBT and 6 months follow-up), treatment, drugs, and parallel depression. For dimensional outcomes (anxiety scores), ITT analyses will be conducted using all participants enrolled in the study, and the last available data point will be used as the post-treatment score. Uncontrolled effect sizes will be calculated for pre- to post-treatment change. For categorical outcomes (adherence), treatments will be compared using Pearson's chi-square test, Fisher's exact test, or logistic regression, as appropriate.

### Ethical considerations

The study protocol was approved by the Regional Research Ethics Committee in Linköping in 2009 (Dnr. 111-09). Before submission to the committee the protocol was reviewed with regard to ethical issues related to the participants, particularly considering non-malfeasance, autonomy, and fidelity [30]. Prior to invitation and randomization, clients displaying signs of severe psychiatric disease are referred to psychiatric specialists. The research will contribute to the development of the evidence base in the area, with potential future benefits for participants and society. All participation will be based on informed consent. The clients will be able to withdraw from the study at any time, while still being supplied routine CBT services. The integrity of the randomization procedure will be checked on a regular basis.

## Discussion

Health care services are increasingly moving out of the physician's office into IT-mediated treatment in the community [18,31]. There is evidence that IT-supported therapy works in controlled settings [20]. However, if evidence is to be translated from stringent RCTs to frontline clinical practice, some obstacles still remain before IT-supported therapy can become routine practice. Two particular differences between evidence from RCTs and frontline clinical settings that need to be investigated are their applicability to total populations and on clients with overt comorbidity. In addition, there are a host of organizational and community context issues that can only be investigated in real-world settings.

Different applications of cell phone support for in situ CBT have recently been reported [32]. The present protocol can be used to study the effectiveness of cell phone-supported CBT for management of anxiety in different practice, organizational, and technical settings. We recognize the lessons learned from early innovative studies that had methodological shortcomings preventing them from transforming from evidence to practice [21]. Although different types of software applications for diagnosis and treatment of anxiety syndromes are available off-the-shelf for stationary computers and the Internet, the availability of high-quality applications for portable computers and cell phones is scarce. To allow the CBT-cell-phone strand to be replicated in other studies, the design of the application used in the case study setting will be described in detail in forthcoming reports.

### Limitations

The study protocol complies with allocation concealment and completeness of follow-up data. However, because of the tangible nature of cell-phone supported therapy, blinding is not supported by the protocol. After having been allocated a client and a treatment in a concealed process, the therapist has total insight in the therapy and, thereby, any use of supportive technologies is not blinded.

## Conclusions

This protocol for use in frontline clinical practice in which effectiveness, adherence, and the role of the therapists are analysed will provide evidence for what are truly valuable cell-phone-supported CBT treatments and guidance for the broader introduction of CBT in health services.

## Competing interests

The authors declare that they have no competing interests.

## Authors' contributions

JE helped in the design of the final study protocol, had part in the technical design, and drafted the initial manuscript; TT conceived the study, designed the final study protocol, had part in the technical design and drafted the initial manuscript; MB drafted the technical design, and designed the final study protocol; AF drafted the technical design; KH designed the final study protocol, HE had part in the technical design and design of the final study protocol. All authors read and approved the final manuscript.

## Pre-publication history

The pre-publication history for this paper can be accessed here:

http://www.biomedcentral.com/1471-2288/11/3/prepub

## References

[B1] WHO The world health report: 2001 - mental health: new understanding, new hope 2001 Geneva: World Health Organization

[B2] Goisman RM Warshaw MG Keller MB Psychosocial treatment prescriptions for generalized anxiety disorder, panic disorder, and social phobia, 1991-1996 Am J Psychiatry 1999 156 1819 1821 10553751 10.1176/ajp.156.11.1819

[B3] Taylor CB Chang VY Issues in the dissemination of cognitive-behavior therapy Nord J Psychiatry 2008 62 Suppl 47 37 44 10.1080/08039480802315673 18752117

[B4] UK Department of Health Improving access to psychological therapies: national guideline for regional delivery 2008 London: Department of Health

[B5] Wang PS Demler O Kessler RC Adequacy of treatment for serious mental illness in the United States Am J Public Health 2002 92 92 98 10.2105/AJPH.92.1.92 11772769 PMC1447396

[B6] Mitte K Meta-analysis of cognitive-behavioral treatments for generalized anxiety disorder: a comparison with pharmacotherapy Psychol Bull 2005 131 785 795 10.1037/0033-2909.131.5.785 16187860

[B7] Butler AC Chapman JE Forman EM Beck AT The empirical status of cognitive-behavioral therapy: a review of meta-analyses Clin Psychol Rev 2006 26 17 31 10.1016/j.cpr.2005.07.003 16199119

[B8] Proudfoot J Goldberg D Mann A Everitt B Marks I Gray JA Computerized, interactive, multimedia cognitive-behavioural program for anxiety and depression in general practice Psychol Med 2003 33 217 227 10.1017/S0033291702007225 12622301

[B9] Marks IM Mataix-Cols D Kenwright M Cameron R Hirsch S Gega L Pragmatic evaluation of computer-aided self-help for anxiety and depression Br J Psychiatry 2003 183 57 65 10.1192/bjp.183.1.57 12835245

[B10] Andersson G Carlbring P Holmstrom A Sparthan E Furmark T Nilsson-Ihrfelt E Buhrman M Ekselius L Internet-based self-help with therapist feedback and in vivo group exposure for social phobia: a randomized controlled trial J Consult Clin Psychol 2006 74 677 686 10.1037/0022-006X.74.4.677 16881775

[B11] Carlbring P Bohman S Brunt S Buhrman M Westling BE Ekselius L Andersson G Remote treatment of panic disorder: a randomized trial of internet-based cognitive behavior therapy supplemented with telephone calls Am J Psychiatry 2006 163 2119 2125 10.1176/appi.ajp.163.12.2119 17151163

[B12] SoS Socialstyrelsen Depressionssjukdom och ångestsyndrom: Vetenskapligt underlag för Nationella riktlinjer 2009

[B13] Kehle SM The effectiveness of cognitive behavioral therapy for generalized anxiety disorder in a frontline service setting Cogn Behav Ther 2008 37 192 198 10.1080/16506070802190262 18608310

[B14] Kopta SM Lueger RJ Saunders SM Howard KI Individual psychotherapy outcome and process research: challenges leading to greater turmoil or a positive transition? Annu Rev Psychol 1999 50 441 469 10.1146/annurev.psych.50.1.441 10074685

[B15] Westen D Novotny CM Thompson-Brenner H The empirical status of empirically supported psychotherapies: assumptions, findings, and reporting in controlled clinical trials Psychol Bull 2004 130 631 663 10.1037/0033-2909.130.4.631 15250817

[B16] Wade WA Treat TA Stuart GL Transporting an empirically supported treatment for panic disorder to a service clinic setting: a benchmarking strategy J Consult Clin Psychol 1998 66 231 239 10.1037/0022-006X.66.2.231 9583326

[B17] Bang M Timpka T Eriksson H Holm E Nordin C Mobile phone computing for in-situ cognitive behavioral therapy Stud Health Technol Inform 2007 129 1078 1082 17911881

[B18] Delbanco T Berwick DM Boufford JI Edgman-Levitan S Ollenschlager G Plamping D Rockefeller RG Healthcare in a land called PeoplePower: nothing about me without me Health Expect 2001 4 144 150 10.1046/j.1369-6513.2001.00145.x 11493320 PMC5060064

[B19] Jones J Hunter D Consensus methods for medical and health services research BMJ 1995 311 376 380 7640549 10.1136/bmj.311.7001.376 PMC2550437

[B20] Cuijpers P Marks I Straten A Cavanagh K Gega L Andersson G Computer-aided psychotherapy for anxiety disorders: a meta-analytic review Cogn Behav Ther 2009 38 66 82 10.1080/16506070802694776 20183688

[B21] Andersson G Carlbring P Berger T Almlov J Cuijpers P What makes internet therapy work? Cogn Behav Ther 2009 38 S1 55 60 10.1080/16506070902916400 19675956

[B22] Almlov J Carlbring P Berger T Cuijpers P Andersson G Therapist factors in internet-delivered cognitive behavioural therapy for major depressive disorder Cogn Behav Ther 2009 38 247 254 10.1080/16506070903116935 19802751

[B23] Eysenbach G The law of attrition J Med Internet Res 2005 7 e11 10.2196/jmir.7.1.e11 15829473 PMC1550631

[B24] Mendel P Meredith LS Schoenbaum M Sherbourne CD Wells KB Interventions in organizational and community context: a framework for building evidence on dissemination and implementation in health services research Adm Policy Ment Health 2008 35 21 37 10.1007/s10488-007-0144-9 17990095 PMC3582701

[B25] Beck AT Steer RA BAI, Beck Anxiety Inventory 1990 San Antonio, TX: Psychological Corporation, Harcourt Brave Jovanovich

[B26] Tait RJ Hulse GK Robertson SI A review of the validity of the General Health Questionnaire in adolescent populations Aust N Z J Psychiatry 2002 36 550 557 10.1046/j.1440-1614.2002.01028.x 12169157

[B27] Bjelland I Dahl AA Haug TT Neckelmann D The validity of the Hospital Anxiety and Depression Scale. An updated literature review J Psychosom Res 2002 52 69 77 10.1016/S0022-3999(01)00296-3 11832252

[B28] Kenardy JA Dow MG Johnston DW Newman MG Thomson A Taylor CB A comparison of delivery methods of cognitive-behavioral therapy for panic disorder: an international multicenter trial J Consult Clin Psychol 2003 71 1068 1075 10.1037/0022-006X.71.6.1068 14622082

[B29] Everitt BS Pickles A Statistical aspects of the design and analysis of clinical trials 1999 London: Imperial College Press

[B30] Heppner PP Wampold BE Kivligan DM Research design in counselling 2008 3 Belmont, CA: Thomson

[B31] Delbanco T Sands DZ Electrons in flight - e-mail between doctors and patients N Engl J Med 2004 350 1705 1707 10.1056/NEJMp038209 15102994

[B32] Morris ME Kathawala Q Leen TK Gorenstein EE Guilak F Labhard M Deleeuw W Mobile therapy: case study evaluations of a cell phone application for emotional self-awareness J Med Internet Res 2010 12 2 e10 10.2196/jmir.1371 20439251 PMC2885784

